# Host-specific exposure and fatal neurologic disease in wild raptors from highly pathogenic avian influenza virus H5N1 during the 2006 outbreak in Germany

**DOI:** 10.1186/s13567-015-0148-5

**Published:** 2015-03-05

**Authors:** Judith MA van den Brand, Oliver Krone, Peter U Wolf, Marco WG van de Bildt, Geert van Amerongen, Albert DME Osterhaus, Thijs Kuiken

**Affiliations:** Department of Viroscience, Erasmus Medical Center, Dr. Molewaterplein 50, 3015 GE Rotterdam, Netherlands; Department Wildlife Diseases, Leibniz Institute for Zoo and Wildlife Research, Alfred-Kowalke-Straße 17, 10315 Berlin, Germany; Department for Diagnostic Investigation of Epizootics (LALLF), State Office for Agriculture, Food Safety, and Fishery, Mecklenburg-Western Pomerania, Rostock, Germany

## Abstract

Raptors may contract highly pathogenic avian influenza virus H5N1 by hunting or scavenging infected prey. However, natural H5N1 infection in raptors is rarely reported. Therefore, we tested raptors found dead during an H5N1 outbreak in wild waterbirds in Mecklenburg-Western Pomerania, Germany, in 2006 for H5N1-associated disease. We tested 624 raptors of nine species—common buzzard (385), Eurasian sparrowhawk (111), common kestrel (38), undetermined species of buzzard (36), white-tailed sea eagle (19), undetermined species of raptor (12), northern goshawk (10), peregrine falcon (6), red kite (3), rough-legged buzzard (3), and western marsh-harrier (1)—for H5N1 infection in tracheal or combined tracheal/cloacal swabs of all birds, and on major tissues of all white-tailed sea eagles. H5N1 infection was detected in two species: common buzzard (12 positive, 3.1%) and peregrine falcon (2 positive, 33.3%). In all necropsied birds (both peregrine falcons and the six freshest common buzzards), H5N1 was found most consistently and at the highest concentration in the brain, and the main H5N1-associated lesion was marked non-suppurative encephalitis. Other H5N1-associated lesions occurred in air sac, lung, oviduct, heart, pancreas, coelomic ganglion, and adrenal gland. Our results show that the main cause of death in H5N1-positive raptors was encephalitis. Our results imply that H5N1 outbreaks in wild waterbirds are more likely to lead to exposure to and mortality from H5N1 in raptors that hunt or scavenge medium-sized birds, such as common buzzards and peregrine falcons, than in raptors that hunt small birds and do not scavenge, such as Eurasian sparrowhawks and common kestrels.

## Introduction

Highly pathogenic avian influenza virus causes high mortality in poultry, but is rarely reported to cause clinical disease in wild waterbirds. However, since 2000 there have been several reported outbreaks of highly pathogenic avian influenza virus of the subtype H5N1 causing mortality in wild waterbirds. A major outbreak occurred in 2005 in western China, causing mortality of more than thousand birds, including bar-headed geese (*Anser indicus*), great black-headed gulls (*Larus ichthyaetus*), and brown-headed gulls (*Larus brunnicephalus*) [1,2]. Subsequently, H5N1 spread to Europe in 2005 and 2006, and caused mortality in a variety of wild birds. In Germany, H5N1 infection was detected by real-time reverse transcriptase polymerase chain reaction (RT-PCR) in several wild bird species, not only waterbirds such as whooper (*Cygnus cygnus*) and mute swans (*Cygnus olor*), Canada geese (*Branta canadensis*), tufted ducks (*Aythya fuligula*), greater black-backed gulls (*Larus marinus*), herring gulls (*Larus argentatus*), but also in raptors such as northern goshawks (*Accipiter gentilis*) and common buzzards (*Buteo buteo*) [3,4]. By histology, these birds showed meningitis and encephalitis, characterized by gliosis and inflammation of the grey matter, neuronal degeneration and necrosis, and perivascular cuffing. These brain lesions were associated with influenza virus antigen expression in neurons and cells of the glia, ependyma and choroid plexus [3]. Additional lesions outside the brain were necrosis in liver, exocrine pancreas, adrenal glands and spleen, and hemorrhage in lungs and heart [3].

Since several raptor species selectively hunt moribund or otherwise conspicuous prey, including waterbirds, or scavenge on their carcasses, they may be assumed to have a high rate of exposure to H5N1 during outbreaks of this virus. However, there are only rare case reports of natural H5N1 infection in raptors: common buzzard [3,5,6], mountain hawk-eagle (*Spizaetus nipalensis*) [7,8], and saker falcon (*Falco cherrug*) [7], with limited information on virus distribution and associated lesions. Therefore, our goal was to determine whether raptors exposed to H5N1 during an outbreak in wild waterbirds suffered fatal disease, and—if so—how the H5N1-associated lesions compared to those in other avian species. To do so, we tested all raptors that were found dead in the region Mecklenburg-Western Pomerania during the H5N1 outbreak in 2006 for H5N1 by RT-PCR of tracheal and/or cloacal swabs, and performed full necropsies on a selection of these birds for the presence of H5N1-associated lesions.

## Material and methods

### Birds

Of the total of 9 356 dead wild birds from Mecklenburg-Western Pomerania examined during the 2006 H5N1 outbreak, 624 were raptors (Tables 1 and 2) [9]. Identification to species was not possible for some carcasses due to advanced state of autolysis. Swabs for virology were collected from each carcass, which was subsequently frozen at −20 °C until potential necropsy. According to the instructions of the Federal Research Institute for Animal Health, National Reference Laboratory for Avian Influenza, Isle of Riems, Germany, combined tracheal and cloacal swabs were taken until 3rd of March 2006: the swab was introduced into the oral cavity to reach the lumen of the trachea, or, when the larynx was too narrow, the throat was cut from outside to take the swab; the same swab was used to sample the cloaca. Beginning 4th of March 2006, only tracheal swabs were taken.

**Table 1:** **RT-PCR results in swabs of wild raptor species found dead in Mecklenburg-Western Pomerania in 2006**

**Species**	**Number of birds**	
	**Tested**	**H5N1-positive (%)**
Common buzzard (*Buteo buteo*)	385	12* (3.1)
Eurasian sparrow hawk (*Accipiter nisus*)	111	0
Common kestrel (*Falco tinnunculus*)	38	0
Buzzard, species undetermined (*Buteo sp*.)	36	1** (2.7)
White-tailed sea eagle (*Haliaeetus albicilla*)	19	0
Raptors, species undetermined	12	0
Northern goshawk (*Accipiter gentilis*)	10	0
Peregrine falcon (*Falco peregrinus*)	6	2*** (33.3)
Red kite (*Milvus milvus*)	3	0
Rough-legged buzzard (*Buteo lagopus*)	3	0
Western marsh harrier (*Circus aeruginosus*)	1	0
Total	624	15 (2.4)

*Seven tested by combined tracheal and cloacal swabs, five tested by tracheal swabs only.

**Tested by combined tracheal and cloacal swab.

***Tested by tracheal swabs only.

**Table 2:** **Sex and age of selected raptor species found dead in Mecklenburg-Western Pomerania in 2006**

**Species**	**Sex**		**Age**		
	**Female**	**Male**	**Juvenile**	**Subadult**	**Adult**
Common buzzard	4	2	2	0	4
Peregrine falcon	2	0	1	0	1
White-tailed sea eagle	12	7	14	2	3

Both selection of carcasses for necropsy and level of sampling of necropsied carcasses differed between white-tailed sea eagles (*Haliaeetus albicilla*) and other raptor species. For white-tailed sea eagles, all 19 individuals underwent necropsy, regardless whether their swabs tested positive or negative for H5N1, and tissue samples of lung, liver, spleen, kidney, pancreas and brain were collected for virology and stored at −70 °C until analysis. Duplicate samples of the same tissues were collected for histopathology and immunohistochemistry and fixed in 10% neutral-buffered formalin until analysis. For other raptor species, only individuals with H5N1-positive swabs and either freshly dead or intact and expected to be in a mild state of autolysis underwent necropsy: six common buzzards (B1 to B6) and two peregrine falcons (*Falco peregrinus*) (F1 and F2). During this necropsy, tissue samples of nasal turbinate, air sac, trachea, bronchus, lung, heart, liver, spleen, kidney, adrenal gland, pancreas, stomach, jejunum, colon, cerebrum, cerebellum, reproductive tract and cloacal bursa (or cloacal area if cloacal bursa was not visible) were collected for virology and stored at −70 °C until analysis. Duplicate samples of the same tissues were collected for histopathology and immunohistochemistry and fixed in 10% neutral-buffered formalin until analysis. In addition, separate pharyngeal and cloacal swabs for virology were placed in Hank’s balanced salt solution containing 0.5% lactalbumin, 10% glycerol, 200 U/mL penicillin, 200 μg/mL streptomycin, 100 U/mL polymyxin B sulfate, 250 μg/mL gentamycin, and 50 U/mL nystatin (ICN Pharmaceuticals, Zoetermeer, The Netherlands), and stored at −70 °C until analysis.

### Virology

Swabs were examined by testing extracted total RNA for the presence of influenza A virus matrix gene-fragment by RT-PCR. A positive result was followed by further subtype differentiation using real-time RT-PCR targeting fragments of the H5 and N1 genes [10]. Tissue samples were weighed and subsequently homogenized with a FastPrep-24 (MP Biomedicals, Eindhoven, The Netherlands) in Hank’s balanced salt solution and centrifuged briefly before dilution. Quadruplicate 10-fold serial dilutions of tissue samples and swab supernatants (separate pharyngeal and cloacal swabs from common buzzards, B1 to B6, and peregrine falcons, F1 and F2, only) were used to determine the virus titers in confluent layers of Madin-Darby canine kidney cells as described previously [11].

### Histopathology and immunohistochemistry

Formalin-fixed tissue samples from common buzzards, B1 to B6, and peregrine falcons, F1 and F2, were embedded in paraffin, sectioned at 4 μm, and stained with hematoxylin and eosin (HE) for histopathological examination by light microscopy. For immunohistochemical detection of influenza virus antigen, duplicate sections of the same tissue samples were stained with a primary antibody against the influenza A nucleoprotein as described previously [12]. Peroxidase activity was revealed using 3-amino-9-ethylcarbazole (AEC) (Sigma, St Louis, MO, USA), resulting in a bright red precipitate. In each staining procedure an isotype control was included as a negative control and a lung section from an H5N1-infected cat was included as a positive control [13].

## Results

### Virology

Out of 624 individual raptors belonging to 9 different species, H5N1 was detected by RT-PCR in the swabs of only 2 species: common buzzard (12 of 385) and peregrine falcon (2 of 6) (Table 1). All pharyngeal swabs and most cloacal swabs of the necropsied common buzzards and peregrine falcons were H5N1-positive by RT-PCR, with comparable viral RNA levels in pharyngeal and cloacal swabs (Table 3). Multiple tissues in the necropsied common buzzards and peregrine falcons were H5N1-positive by RT-PCR (Table 3) in contrast to tissues of the 19 white-tailed sea eagles, all of which were H5N1-negative. Overall, the viral RNA levels in the brain samples of both common buzzards and peregrine falcons were considerably higher than in any other tissue samples. Interestingly, viral RNA levels were high in pancreas samples of the peregrine falcons but not in those of the common buzzards.

**Table 3:** **RT-PCR results and virus titers in swabs and organs of selected H5N1-infected wild raptors**

RT-PCR values* (virus titers)**								
**Samples**	**Common buzzards**						**Peregrine falcons**	
	**B1**	**B2**	**B3**	**B4**	**B5**	**B6**	**F1**	**F2**
Pharyngeal swab	24.2 (2.2)	26.7 (0.8)	23.9 (0.8)	26.9 (n)	29.0 (1.2)	28.9 (n)	26.8 (2.2)	28.8 (n)
Cloacal swab	25.9 (n***)	np****	n (n)	24.6 (n)	28.2 (n)	31.0 (n)	16.5 (2.5)	28.2 (1.5)
Lung	18.1 (5.1)	19.6 (5.0)	17.6 (2.8)	14.4 (2.0)	22.0 (4.2)	24.5 (3.4)	22.1 (2.8)	34.6 (4.0)
Air sac	17.3 (n)	19.2 (2.6)	21.2 (n)	21.0 (n)	17.5 (2.5)	22.2 (n)	13.2 (n)	25.9 (n)
Liver	n (n)	28.3 (n)	26.1 (n)	21.9 (n)	24.5 (n)	20.7 (n)	20.3 (n)	21.6 (n)
Spleen	21.5 (n)	21.6 (n)	25.5 (n)	27.5 (n)	28.4 (n)	22.1 (n)	26.7 (n)	21.2 (n)
Heart	24.0 (n)	21.9 (2.3)	17.4 (n)	19.6 (n)	15.3 (n)	17.2 (n)	20.1 (n)	19.5 (n)
Kidney	24.5 (1.6)	23.2 (n)	19.7 (n)	15.0 (n)	19.5 (n)	19.9 (n)	18.4 (n)	26.4 (n)
Jejunum	18.9 (1.9)	17.7 (n)	29.7 (n)	29.3 (n)	22.6 (n)	20.7 (n)	21.8 (n)	22.7 (n)
Colon	20.4 (n)	21.4 (n)	29.6 (n)	27.1 (n)	21.4 (n)	19.7 (n)	24.4 (n)	22.4 (n)
Pancreas	26.7 (n)	25.0 (n)	38.0 (n)	34.7 (n)	29.8 (n)	34.3 (n)	9.6 (n)	13.7 (2.5)
Cloaca/Bursa	np	21.4 (n)	29.1 (2.1)	20.8 (n)	np	np	np	20.9 (n)
Brain	15.5 (6.2)	9.5 (6.3)	13.4 (5.5)	11.3 (6.7)	9.1 (6.2)	18.5 (5.2)	8.8 (4.6)	14.1 (6.3)

*Cycle threshold (Ct) value: The cut-off value is 40.

**All titers are given in TCID_50_ log 10.

***n = negative (<0.5 for virus titers).

****np = not present.

By virus culture, pharyngeal swabs of necropsied common buzzards and peregrine falcons tested positive for H5N1 more frequently than cloacal swabs (Table 3). Far fewer tissue samples tested positive for H5N1 by virus culture than by RT-PCR (Table 3): H5N1 was consistently cultured only from brain and lung samples in both common buzzards and peregrine falcons, always with the highest titers in brain samples.

### Gross pathology

The nutritional state of the white-tailed sea eagles ranged from poor to good. All common buzzards and peregrine falcons were in poor nutritional state, lacking fat in the subcutis or body cavity, and showing mild to advanced atrophy of the breast muscles. Externally, no lesions were found. Internally, there were lesions in several organs: multifocal hemorrhage in the pancreas (B2 and B3); increased redness in the cerebrum (B1, B2, B3, B5, and F2); enlargement of the spleen (B1, B2 and B6) and kidneys (B1, B2, and B5); wet lungs (B4, B5, B6 and F2); many nematodes (most likely *Serratospiculum sp.*) in the abdominal air sac (F1 and F2), and multifocal hemorrhage in thorax or upper respiratory tract, suggestive of trauma (F1 and F2). Freeze-thaw artifacts and autolytic changes often hampered gross examination.

### Histopathology

**Figure 1:**
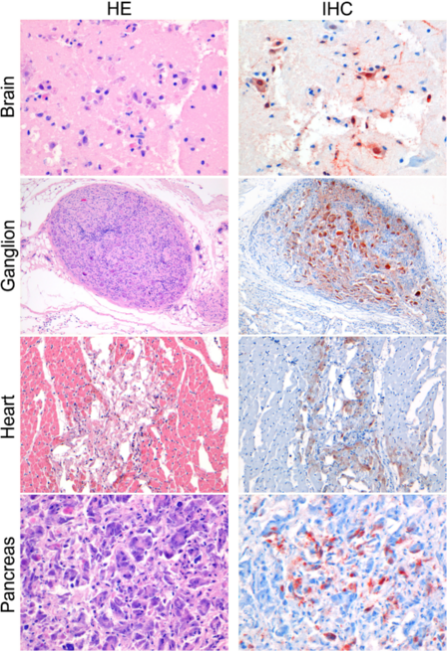
**Histopathological changes and influenza virus antigen expression in tissues of selected H5N1-infected wild raptors.** Tissue sections in the left column are stained with hematoxylin and eosin (HE). Serial tissue sections in the right column are stained for influenza virus antigen by immunohistochemistry (IHC). In the brain (F1) and coelomic ganglion (B4), there is neuronal necrosis and lymphoplasmacytic and histiocytic inflammation associated with antigen expression in neurons and glial cells (red-brown staining). In the heart (B2) and pancreas (F1), there is necrosis and lymphoplasmacytic and histiocytic inflammation associated with antigen expression in the cardiomyocytes (heart) and exocrine pancreatic cells (pancreas). In all selected tissues freeze-thawn artifacts and mild autolytic changes are visible.

By histopathology, lesions were seen in the brain, air sac, oviduct, coelomic ganglion, heart, pancreas and adrenal gland of common buzzards and peregrine falcons (Figure 1). In the cerebrum and occasionally in the cerebellum, there was multifocal necrosis of neurons with hypereosinophilia of the cytoplasm, nucleomegaly, neuronophagy and mild increase of glial cells. Separately from this neuronal necrosis, there was a multifocal increase of glial cells with mild to moderate diffuse or perivascular infiltration with lymphocytes, plasma cells and macrophages. Furthermore, in the brain of one peregrine falcon (F2), there was multifocal mild to moderate infiltration with mononuclear cells around blood vessels, in meninges and beneath the ependyma, with necrosis of ependymal cells. In the coelomic ganglion (B4 and F2), there was multifocal necrosis of neurons with hypereosinophilia of cytoplasm, nucleomegaly, neuronophagy and mild infiltration with mononuclear cells. In the lamina propria of the air sac of almost all birds (not B4), there was necrosis of epithelial cells and a mild to moderate infiltration with mononuclear cells. In the lamina propria of the oviduct (B1 and F1), there was moderate infiltration with mononuclear cells. In the heart (B1, B2, B4 and B6), there was multifocal necrosis of cardiomyocytes and mild infiltration with mononuclear cells. In the adrenal gland of one common buzzard (B5), there were multiple large areas with central core of necrosis surrounded by a layer of macrophages, heterophils, plasma cells and lymphocytes. In the pancreas (F1 and F2), there was multifocal necrosis of pancreatic acinar cells and marked infiltration with mononuclear cells. In the bronchus (F2) and parabronchus (B1, F1 and F2) in the lungs of few birds there was mild infiltration on predominantly heterophils in the epithelium and interstitium and mild epithelial necrosis. In the parabronchus and air sac of the peregrine falcons (F1 and F2), there were also intralesional helminth eggs in the lumina and in the epithelium (consistent with *Serratospiculum sp.* infection). Freeze-thaw artifacts and mild autolytic changes were present in most tissues, but did not prevent histopathological assessment.

### Immunohistochemistry

By immunohistochemistry, influenza virus antigen expression was visible as diffuse red staining mainly in the cytoplasm and occasionally in the nucleus of affected cells. Virus antigen expression was found in multiple tissues (Figure 1) and was associated with histopathological changes. Virus antigen expression was seen most frequently in the brain (all birds) and air sac (B1-3, B5, B6 and F1). In the brain there were multiple foci of positive cells in the cerebrum of all common buzzards and peregrine falcons and in the cerebellum of some birds. In the brain, many neurons and—in one peregrine falcon, F2—ependymal cells, and few glial cells expressed virus antigen. In the coelomic ganglion (B4 and F2), many neurons expressed virus antigen. In the air sac, oviduct (B1, B2 and F1), and bronchus (F2), few epithelial cells expressed virus antigen. In the heart (B2, B4 and B5), few myocardial cells expressed antigen. In multiple granulomas in the adrenal gland of one common buzzard (B5), many macrophages expressed antigen. In the pancreas (F1 and F2), moderate numbers of pancreatic acinar cells expressed antigen.

## Discussion

Our study shows that, during the 2006 H5N1 outbreak in wild waterbirds in Mecklenburg- Western Pomerania, Germany, both common buzzards and peregrine falcons were exposed to and suffered fatal disease from H5N1 infection, while seven other raptor species examined showed no evidence of H5N1 infection. In both species, H5N1 showed a distinct neurotropism associated with marked encephalitis, comparable to that observed in most other susceptible wild bird species.

The differences in diet and hunting or scavenging behavior among the nine raptor species was likely an important factor in their exposure to H5N1 in the 2006 outbreak in Germany. This outbreak affected mainly swans, geese, gulls, and diving ducks, all medium-sized birds [14] and the viruses of the waterbirds were closely related to the virus found in a common buzzard that tested positive for H5N1 in the same period and the same area [4]. The Eurasian sparrowhawk and common kestrel do not eat carrion and hunt only small (passerine) birds [15,16] or small mammals [17]; this could explain why H5N1 was not detected in either raptor species, despite the relatively high number of individuals examined. In contrast, the common buzzard and peregrine falcon, both of which were found positive for H5N1, do hunt medium-sized birds—common buzzards up to the size of a pheasant (~1200 g) [15]; peregrine falcons mainly in the range 20 to 300 g for males, and 100–1000 g for females [18,19]—or feed on carrion (common buzzard) [20]. (Interestingly, both H5N1-positive peregrine falcons were females.) The remaining five raptor species (white-tailed sea eagle, northern goshawk, red kite, rough-legged buzzard, and western marsh-harrier) all are known to hunt medium-sized birds and—except for the northern goshawk—to eat carrion, and therefore were at risk of exposure to H5N1 during the outbreak. Remarkably, the white-tailed sea eagles which feed on waterbirds up to the size of swans and also on carrion, were tested negative. The reason why H5N1 was not detected in any individuals of these five species may be due to the small sample size. Alternatively, it may be due to a species-specific resistance to disease from H5N1 infection, similar to that seen in other avian species. For example, in experimental H5N1 infection of six different duck species, tufted ducks (*Aythya fuligula*) and Eurasian pochards (*A. ferina*) showed clinical signs of disease, while the remaining four species, all dabbling ducks belonging to the genus *Anas*, were clinically unaffected [21].

The tissue tropism of H5N1 and the character of the associated lesions in these common buzzards and peregrine falcons correspond with those seen previously in other avian species (reviewed in [22,23], including naturally [3,5,6,24] and experimentally [25,26] H5N1 infected raptors. H5N1 in these common buzzards and peregrine falcons was clearly neurotropic and associated with marked encephalitis, similar to that seen in naturally infected common buzzards from Germany [3] and Sweden [5] and in experimentally infected American kestrels (*Falco sparverius*) [25]. The detection of influenza virus antigen expression and associated lesions in the coelomic ganglion in our birds indicates neurotropism for both the peripheral and central nervous systems. Besides the nervous system, H5N1 in our common buzzards and peregrine falcons also had tropism for other organs, including pancreas, heart, lung, air sac and oviduct. Such pantropism corresponds with that found in other bird species (reviewed in [22,23]. The specific tropism of H5N1 for the pancreas in our peregrine falcons also has been noted in experimental H5N1 infection of American kestrels [25]. In contrast to different swan species, where H5N1 exhibited endotheliotropism [3], no influenza virus antigen expression was seen in endothelial cells of any of these common buzzards or peregrine falcons.

The discrepancy between the results in the immunohistochemistry, RT-PCR and virus titration can most likely be attributed to the extended storage time in the −20 °C freezer and to the difference in sensitivity of the tests. Also, autolysis before storage and freezing artifacts hampered the necropsy and data analyses.

Although it is likely that these common buzzards and peregrine falcons became infected with H5N1 because of feeding on H5N1-contaminated carcasses, as has been shown experimentally in gulls [27], it is not clear by which of three potential portals of entry H5N1 gained entrance. The first possible portal of entry is the intestine, as proven by direct gastric inoculation of H5N1 in different experimental mammals [28-31]. A second possible portal of entry is the olfactory nerve: this was proven by intranasal inoculation of H5N1 in ferrets, in which viral replication in the olfactory mucosa was followed by spread to the olfactory bulb, and from there to the rest of the central nervous system [32]. The third possible portal of entry is the respiratory tract. Because H5N1-contaminated meat in these common buzzards and peregrine falcons would have passed the throat, which is the cross-roads between digestive and respiratory tracts, this latter portal of entry cannot be excluded. More extensive sampling of H5N1-infected raptors at different time points after infection may elucidate the portal of entry and subsequent pathogenesis of H5N1 infection in raptors.

In conclusion, our study implies that the position of raptors at the top of the food pyramid puts them at risk of acquiring infectious pathogens such as H5N1 by eating sick or dead prey. Because of their broad geographic range and migratory nature, they may contribute to long-distance movement of such pathogens; however, this contribution is likely much smaller than that of more numerous species of migratory birds. Our data suggest that some raptor species, such as common buzzards and peregrine falcons, are more likely to eat sick or dead wild waterbirds and subsequently are more exposed to H5N1 infection than other species, such as Eurasian sparrowhawks and common kestrels. Whether there is species-specific resistance to disease from H5N1 infection in some raptor species, such as white-tailed sea eagles, remains to be determined.
